# Interkingdom and intrakingdom interactions in the microbiome of *Heterobasidion* fruiting body and associated decayed woody tissues

**DOI:** 10.1128/aem.01406-23

**Published:** 2023-11-28

**Authors:** Wenzi Ren, Reijo Penttilä, Risto Kasanen, Fred O. Asiegbu

**Affiliations:** 1 Department of Forest Sciences, University of Helsinki, Helsinki, Finland; 2 Natural Resources Institute Finland (Luke), Helsinki, Finland; University of Illinois Urbana-Champaign, Urbana, Illinois, USA

**Keywords:** *Heterobasidion*, interkingdom, intrakingdom, microbiome interaction, wood decay process

## Abstract

The *Heterobasidion annosum* species complex is a major threat to the forest industry in the northern temperate regions of the world. The fungal and bacterial biota communities in *Heterobasidion*-infected trees have been extensively studied, but less is known about the interkingdom and intrakingdom interactions among the microbiome. The bacteria-fungi interkingdom analysis has revealed significant findings compared to the intrakingdom analysis in recent studies. In this study, we aimed to (i) unravel the contribution and development of the bacteria and other fungi in the *Heterobasidion*-infected wood decay process; (ii) uncover how environmental factors and the conifer pathogen drive the microbiome community structure and function changes; and (iii) identify the core microbiome during the *Heterobasidion*-mediated wood decay process. Internal transcribed spacer and 16S amplicon sequencing data from the *Heterobasidion* fruiting body and its associated woody tissue were applied in this analysis. Samples collected from managed forest and nature reserve forest were classified into four decay classes based on the extent of decay of the wood. Our result showed that bacteria and fungi have different abundances and activities in both study materials. The *Heterobasidion* ecological correlation network is conserved, especially in the fruiting body. The core microbiome defined in our analysis showed huge potential for the microbiome community’s functioning. A particular *Heterobasidion* Operational Taxonomic Units (OTU) had the most important impact on the community and disease development. Forest management has the biggest impact on the microbiome assembly, and the fungal community is more sensitive to environmental changes.

**IMPORTANCE:**

We applied macro- (forest stand and forest management) and micro-scale (bacterial and fungal community) analyses for a better understanding of the *Heterobasidion* pathosystem and associated wood decay process. The core microbiome, as defined by hierarchy analysis and a consistent model, and environmental factors correlation with the community assembly were found to be novel.

## INTRODUCTION

The *Heterobasidion annosum* species complex has been a major threat to the forest industry for over a century. Conifers in Eurasia and North America experience huge economic losses annually because of root and stem rot diseases caused by the pathogen (1). The *Heterobasidion annosum* (Fr.) Bref. *sensu lato* (s.l.) species complex has three native species in Europe (2), two species in North America, and one species in Asia (3, 4). The Anthropocene softwood requirement accelerated the spread and development of *Heterobasidion* root rot disease, as wounded wood tissues provide exposure for spore colonization (5). Most efforts have been devoted to balancing industry requirements and disease development. Silviculture, chemical control, and biological control are the three main methods (6–8) currently used for disease management. Considering the cost efficiency, an environmentally friendly, biological control approach has been recognized as the best option to manage the disease. However, the diverse lifestyles and invisible symptoms in the early infection period make it difficult and complicate *Heterobasidion* disease control management (2). The coevolution of tree pathogens and biocontrol agents makes it urgent to find potential new control agents (8–10).

The virulence of pathogens and the resistance levels of plants are the initial basal factors for many tree diseases, while the environment influences the success rate of the infection. Microorganisms usually interact with all the participants and with each other, and the combined outcome will eventually influence the development of the disease (11). The outcome of microbiome interactions could be either mutualism, parasitism, or neutralism (12). A better understanding of the composition, structure, and function of microorganisms is often crucial to helping uncover the mechanism of the disease.

In recent years, the bacterial biota and mycobiome of *Heterobasidion* infection-related tissues have been studied to help understand the infection process, resistance biology, and biocontrol agent detection. Previous studies have reported on the bacterial and fungal composition and structure of *Heterobasidion*-infected wood and the influence of *Phlebiopsis gigantea* application on the communities (13, 14). Community differences between asymptomatic and symptomatic *Heterobasidion*-infected plant anatomies have also been studied (15, 16). Antagonistic agents against *Heterobasidion* have also been broadly identified, providing microbial and mycobiota perspectives (17, 18). However, most of the studies have shown the fragment of a single kingdom in the decay process, which might not be a true reflection of the full dynamics of the interaction within the microbiome community assembly.

Interkingdom interactions are expected to provide a crucial perspective on our understanding of the disease process and microbiome interactions. The interactome will highlight useful insights into the complex representation of functional interactions between fungal and bacterial biota to explain community structure and assembly (19). In principle, taking into account all the organisms associated with the pathobiome would most likely facilitate the construction of a disease model. Many recent studies have included bacteria and fungi in joint analysis, thereby revealing significant impacts on the microbial community, which was not possible with single-kingdom analysis. Gao et al. (20) revealed the complexity of inter- and intrakingdom co-occurrence networks in response to drought stress in soil, roots, the rhizosphere, and leaves. Wagg et al. (21) proved that ecosystem functioning could be predicted by interkingdom and intrakingdom diversity and co-occurrence networks. Durán et al. (22) found that interkingdom interactions in roots promote *Arabidopsis thaliana* survival.

Environmental factors, as key parameters influencing disease, are also able to drive the assembly and development of bacterial and fungal communities (11). Moisture is one of the most important factors influencing fruiting body formation and development (23). Under unfavorable conditions, fewer fruiting bodies and spores form; consequently, for diseases that are spread via spores, a lower spore density is likely to limit the efficiency of disease primary infection. Primary infection is one of the most important ways for *Heterobasidion* to spread during timber harvest seasons (24). Temperature is also significant for the bacterial and fungal communities. For example, temperature influences the activeness of bacteria and fungi; moreover, it could influence the robustness of plants. A higher robustness of the plant and a lower activeness of the pathogen would restrict the disease spread (11, 25). Soil type and soil organic and abiotic contents also influence microbiota assembly and plant growth (26, 27).

Several ecological factors have been reported to impact the *Heterobasidion* pathosystem. Soil type (28), climate (29), management (30, 31), and stand structure (32) have all been previously proposed to influence *Heterobasidion* disease incidence and development. However, most studies have directly correlated the parameters between environmental factors and disease, but fewer studies have reported on the correlation between environmental factors and specific microbial community indicators. Furthermore, although pathogen virulence has been extensively discussed in *Heterobasidion* pathosystems, the focus has primarily been on specific *Heterobasidion* isolates (33, 34). This apparently does not shed light on the totality of the inter- and intrainteractions between all the microbiota in the system.

The purpose of this study is to (i) unravel the important kingdom in the microbiota of *Heterobasidion-*infected wood decay process and fruiting body decay process through community composition, structure, and function perspectives; (ii) uncover the correlations between outer environmental and inner microbiota factors that drive the community structure and functions; and (iii) discuss the keystone microbiota development in the decay process, including *Heterobasidion*. Our hypothesis is that (i) bacterial and diverse fungal species have diverse functional roles in the *Heterobasidion* wood decay process, and (ii) environmental factors and *Heterobasidion* are driving factors for community development in the decay process.

## MATERIALS AND METHODS

### Sampling background

Samples in this study were collected from six Norway spruce-dominated stands in southern Finland. Three of them were managed forests, the purpose of which was timber harvest, and they were from Lapinjärvi, Myrskylä, and Sipoo. The others were from stands with nature conservation or recreational purposes; they were from Lapinjärvi (nature conservation side), Sipoo (nature conservation side), and Viikki. Thirty-eight collection points were found with woody tissue attached to or associated with *Heterobasidion* fruiting bodies, and 76 samples were collected in total, half from fruiting bodies and half from wood. Visual morphological analysis and further internal transcribed spacer (ITS)-based analysis confirmed that the collected fruiting bodies were *Heterobasidion* spp. and that the adhering wood had been infected. *Heterobasidion* fruiting bodies and woody tissue with attached fruiting bodies were collected. Detailed information on each collection point can be found in a previous study (35). The collected fruiting body and wood were classified into four decay classes based on the wood decay status (36). The four decay classes were as follows: D1) recently dead wood, which means that wood is still hard, knife blade can penetrate a few millimeter, and the bark is normally intact; D2) weakly decayed wood, which means that the outer layer of wood stem has started to soften, the wood is still fairly hard, the knife blade is able to penetrate <2 cm, and the bark is loose; D3) medium decayed wood has the outer layer of stem fairly soft; however, the core is still hard, the knife blade penetrates 2–5 cm, and usually no bark is attached; and D4) highly decayed wood, where the wood is soft throughout the log, with no hard core, and the knife blade penetrates all the way. There were 16, 8, 5, and 13 collection points found for wood decay classes D1 to D4, respectively. Fruiting bodies (F) and wood (W) from the four decay classes were represented by D1F-D4F and D1W-D4W, respectively.

The wood samples were obtained from stumps, logs, and standing trees. The stand age varied from 0 to 109 years, and the canopy cover varied from 0 to 71%. The diameter at breast height (DBH) varied from 14 to 40 cm (e.g., for the stump, which was lower than 1.3 m above ground height, it was measured at the highest part). Stand age and canopy cover data were collected from the National Land Survey of Finland (https://kartta.paikkatietoikkuna.fi/). Samples were stored in a cooling box before being transferred into a −20°C environment. Samples were collected in June 2020.

### DNA extraction, amplification of the 16S and ITS rDNA regions, and sequencing

DNA extraction, amplicon amplification, and sequencing were performed as described in a previous study (35). Briefly, samples were pretreated with running water, immersed in 70% EtOH for 1 minute, immersed in 1% commercial bleach for 1 minute, washed with sterile distilled water, and dried with a paper towel. Samples were then homogenized after freeze-drying (37). DNA was extracted by the modified (38) standard cetyltrimethylammonium bromide method (39). DNA quality and purity were checked by a NanoDrop ND-1000 spectrophotometer (Thermo Fisher Scientific, USA). PCR amplification and sequencing of the fungal ITS2 region and bacterial 16S rDNA V3–V4 region were performed at Novogene (Cambridge Science Park, UK). The PCR products were purified and sequenced with the Illumina platform. The fungal amplicon primers were ITS3-2024F 5′-GCATCGATGAAGAACGCAGC-3′ and ITS4-2409R 5′-TCCTCCGCTTATTGATATGC-3′; the bacterial primers were 341F 5′-CCTAYGGGRBGCASCAG-3′ and 806R 5′-GGACTACNNGGGTATCTAAT-3′. Paired-end raw reads (250 bp) were generated for each sequence. Raw sequences obtained in this study are available from the Sequence Read Archive of the National Center for Biotechnology Information (NCBI) under project number PRJNA800074 for bacteria and under project number PRJNA992966 for fungi.

### Bioinformatics

The bioinformatics details are as previously reported (35; Ren et al., unpublished data). Briefly, the raw 16S rRNA sequences and ITS2 rRNA sequences with barcodes and primers were removed at Novogene, UK. For fungal sequences, the QIIME2 pipeline was used to merge paired-end reads after quality control, denoising, and chimeric removal. Quality control was performed in DADA2 with the principle that both directions of sequences held over 25 quality scores (40). The taxonomic references were BLASTed in UNITE and INSD full data sets (41). Singleton Operational Taxonomic Units (OTUs) and OTUs with fewer than 10 reads were removed, and OTUs that were not identified as fungi were removed as well. For bacterial sequences, FLASH Version 1.2.7 was used to merge paired-end reads (42). The QIIME pipeline was used to filter the high-quality data (43). OTUs were created based on the sequences with ≥97% similarity (44). Taxonomic groups were assigned by referencing the SSUrRNA database of the SILVA database using Mothur software (45). In contrast to a previous study, OTUs with unidentified phyla were removed. Finally, 64 fruiting body and wood samples had high-quality fungal and bacterial data.

### Statistical analysis

Normalization was conducted before further analysis. Fungi contained 7,807,417 sequences in total, and each sample contained between 31,664 and 119,314 sequences. Bacteria contained 5,921,960 sequences in total, ranging from 42,217 to 114,758 per sample. The smallest sample sizes of 31,664 and 42,217 were used for normalization. Finally, 3,347 and 7,462 OTUs were obtained from fungi and bacteria, respectively. The Shannon diversity index, species richness, and Pielou’s evenness were chosen to represent fungal community diversity, richness, and evenness; Shannon diversity, Chao1, and Shannon evenness were selected for the bacterial community. Functions of communities were identified using FUNGuild and FAPROTAX for fungi and bacteria (46, 47).

For network analysis, OTUs with over 0.1% relative abundance were selected. The correlations with Spearman correlation values >0.8 and *P* < 0.001 were selected to visualize the result by Gephi (Version 0.9.2). The degree distribution and number of edges were calculated in Gephi and visualized in R Studio (version 4.2.0). Keystone OTUs were selected based on OTUs that had the top 10% in the network. The hierarchy networks were performed by Cytoscape (version 3.9.1), where *Heterobasidion* OTUs were defined as the first level of hierarchy, and OTUs that were directly connected to *Heterobasidion* were defined as the second level. The developing model was built based on the principle of constancy of OTUs in the community (Ren et al., unpublished data). Basically, the model includes constant OTUs, opportunistic OTUs, and other OTUs. The constant OTUs show up in all decay classes of the network, and the opportunistic OTUs only stay in the particular decay class when they show up. The Mantel test was analyzed and visualized by R with Rstudio (48; version 4.2.0). All the alpha diversities for bacteria and fungi, functions that had significant changes or dominant status, and fungal-bacterial communities were used to calculate the Euclidean distance with all environmental factors. Dominating in community function and structure, *Heterobasidion* OTUs were considered one environmental factor, and their OTU number was used to correlate with community values. Decay classes in the environmental data were marked by 1–4, where a larger number represented more significant decay. The study materials were marked by “1” for fruiting body and “2” for wood. As the canopy cover of forest was recorded as a range in the online data set (National Land Survey of Finland, https://kartta.paikkatietoikkuna.fi/), the smallest value of the range was selected. The age of stand was classified into three levels: young, mature, and old, according to a previous study (35). Managed forest was represented by “50,” and nature conservation forest was represented by “1” to represent the stand management intensity. The metadata for detailed environmental parameters can be found in Table S1.

## RESULTS

### Interkingdom and intrakingdom correlations

**FIG 1:**
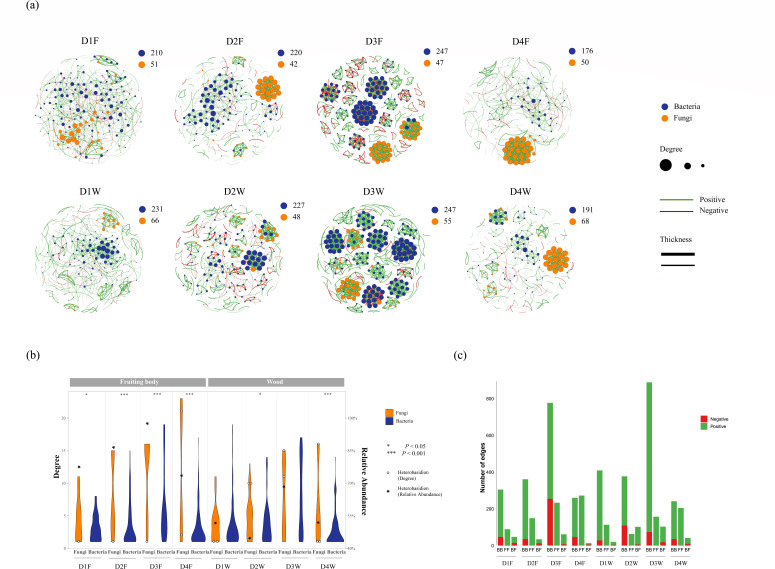
Inter- and intrakingdom networks and the associated topology features. (**a**) Interkingdom network in fruiting bodies and wood of decay classes. Each node represents one OTU; the blue node represents bacterial OTUs; and the orange node represents fungal OTUs. Bacterial and fungal OTU numbers were indicated in the right-top corner of each. The degree of the node represents the connection number with other nodes; a higher degree level indicates that the node has more connections with others. The edge between nodes means their connection; the green edge represents a positive connection, while the red edge represents a negative connection. The thickness of the edge represents the correlation level; a thicker edge indicates a higher correlation level (Spearman correlation values). (**b**) Degree distribution of bacteria and fungi in the study materials. *Heterobasidion* degree distribution and relative abundance were also indicated by circle and star symbols. The relative abundance of *Heterobasidion* was based on only fungal OTUs. (**c**) Degree numbers of positive and negative correlation between inter- and intra kingdom correlation.

The interkingdom and intrakingdom correlations highlight interactions between bacteria and fungi and within the kingdoms. They also reveal the activeness of bacteria and fungi in the community. In the interkingdom correlation network, we found that only 18.4% ± 3.0% of nodes belonged to fungi in the fruiting body, and 21.0% ± 4.1% belonged to fungi in wood (mean ± SE; Fig. 1a). As shown in Fig. 1a, the D3 class had the most active and modulated network interaction, and the D1 class had the least in both materials. The overall interactions of the community and each bacterial OTU degree reached their peak at the D3 class; interactions between each bacterial OTU and others were found to be the most abundant. Interactions between each fungal OTU and others were the second most abundant in fruiting bodies and wood at this stage (Fig. 1b and c). Bacteria gained the most abundant OTUs in the D3 class and the least in the D4 class in both fruiting bodies and wood. Fungi gained the highest OTUs in D1 and D4 but the least in D2 for both fruiting bodies and wood. Modules started to be constructed in the D2 class and developed most abundantly in the D3 class, and finally, a fungal module was constructed in the D4 class of both fruiting bodies and wood (Fig. 1a). The fruiting bodies first formed a fungal module in the D2 class, while wood first formed a bacterial module in the D2 class. Bacterial modules were more abundant than fungal modules in the D3 class of both materials; however, wood gained more bacterial modules than fruiting bodies. Finally, a fungal module was constructed for both fruiting bodies and wood in the D4 class.

The quantitative comparison of the network activity and connection properties between bacteria and fungi showed further information between fungi and bacteria (Fig. 1b). First, the maximum fungal degree tended to increase in both materials in the decay process, while bacteria did not, with fluctuations, especially in wood (Fig. 1b). Even though bacteria were more abundant than fungi in all conditions (Fig. 1a), their degree distribution accumulated to a low value (Fig. 1b). The fungal degree distributions were also more even, especially in fruiting bodies (Fig. 1b). This indicated that a small number of fungal OTUs tended to have more connections in the whole decay process, while the larger OTU participation of bacteria only stayed at a low connection level.

Some of the *Heterobasidion* OTUs showed the same developing trend of top fungal degree distribution in the wood decay process, as they both increased from D1W to D4W. However, this was not obvious in fruiting bodies, where four out of five *Heterobasidion* OTUs were distributed at the bottom area of the degree distribution (Fig. 1b). The relative abundance of *Heterobasidion* presented the same developing trend as the increment of the top fungal degree distribution from D1F to D3F; however, the top fungal degree continued to increase in D4F, while *Heterobasidion* relative abundance in D4F dropped to levels lower than those in D1F. *Heterobasidion* relative abundance fluctuated in wood, and no correlation with fungal degree distribution could be observed (Fig. 1b). Interestingly, the relative abundance of *Heterobasidion* had the same developing trend as the active number of bacteria in the decay process of wood and fruiting bodies (Fig. 1a), and the same trend was also found with the bacteria-bacteria interacting edges and the overall edges in the decay process of both materials (Fig. 1c).

The OTU connection property was different between the fruiting body and wood (Fig. 1c). Interkingdom (bacteria–fungi) interactions appear to be less common than intrakingdom (bacteria–bacteria and fungi–fungi) interactions in nature. Moreover, bacteria–bacteria interactions were the most abundant. Noticeably, fungal–fungal edges continued to increase from D1F to D4F and gained more edges than bacterial–bacterial interactions in the D4 class. However, it decreased from D1W to D2W and increased from D2W to D4W. Bacteria–bacteria and fungi–fungi tended to have the same number of edges in the D4 class of both materials. The interkingdom interactions had the fewest edges compared to the intrakingdom interactions in all fruiting body decay classes and decay classes except D2W. Most negative interactions were found in bacteria-bacteria correlation, few in bacteria-fungi, and only two in fungi-fungi (one in D1F and one in D4W; Table S2). In the interkingdom and intrakingdom correlations, a positive correlation means that these OTUs are ecologically positively correlated with each other, have similar niches, or are mutualistic. However, a negative correlation indicates that the OTUs are antagonistic to each other or that they have competing relationships.

### Keystone OTUs in the interkingdom network

**FIG 2:**
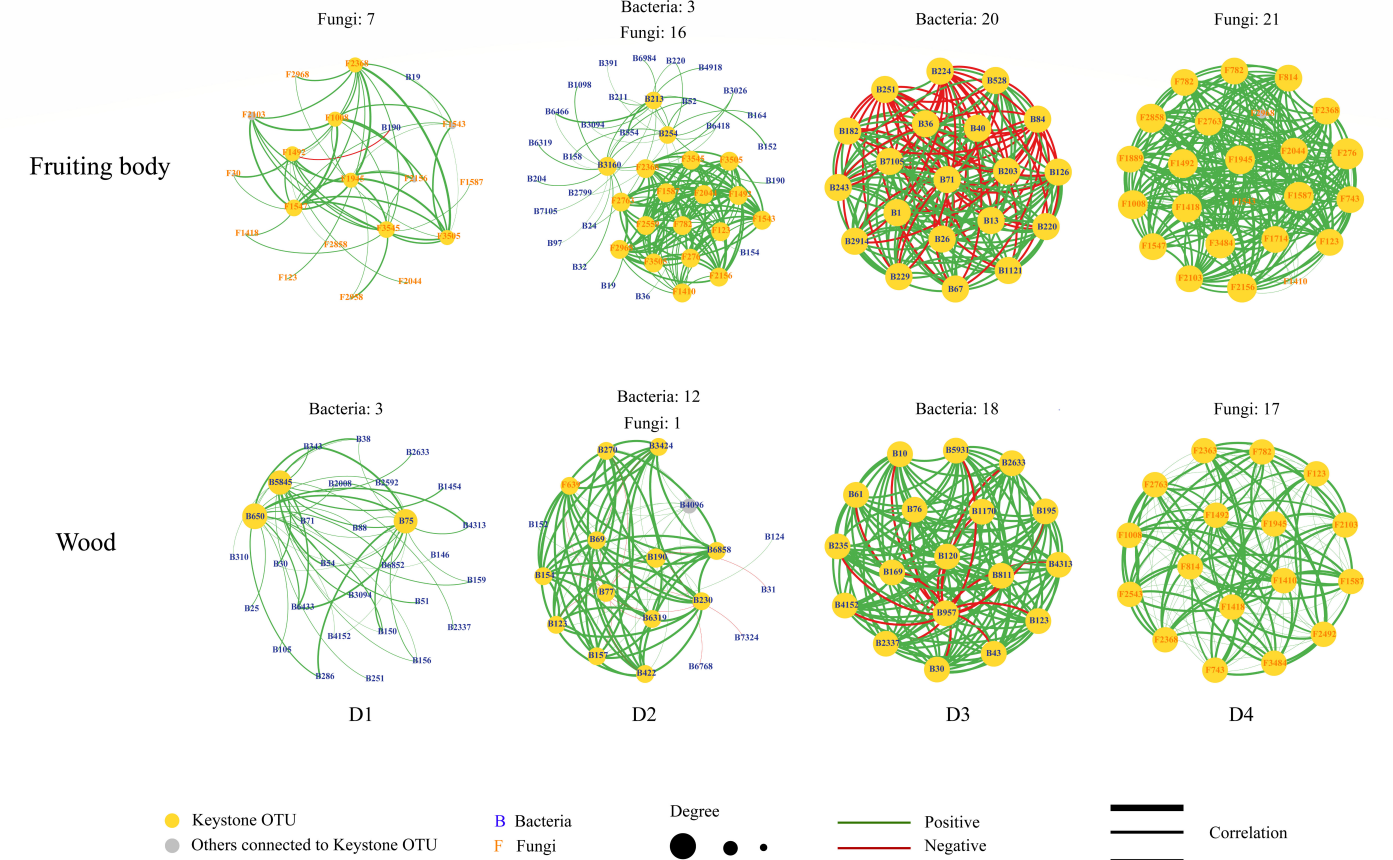
Keystone OTU networks, including keystone OTUs and the other OTUs directly connected.

Keystone OTUs depicted the most active OTUs in the community, and the dominant kingdoms of keystone OTUs and the development of the keystone OTU network differed between fruiting bodies and wood (Fig. 2; Table S3). The microbiome community was dominated by different kingdoms at the beginning of decay in different host materials; fungi dominated in the fruiting bodies and bacteria dominated in wood, while they developed in the same direction as the fungi-dominated keystone network at the end of decay (Fig. 2).

### Interkingdom hierarchy connections

**FIG 3:**
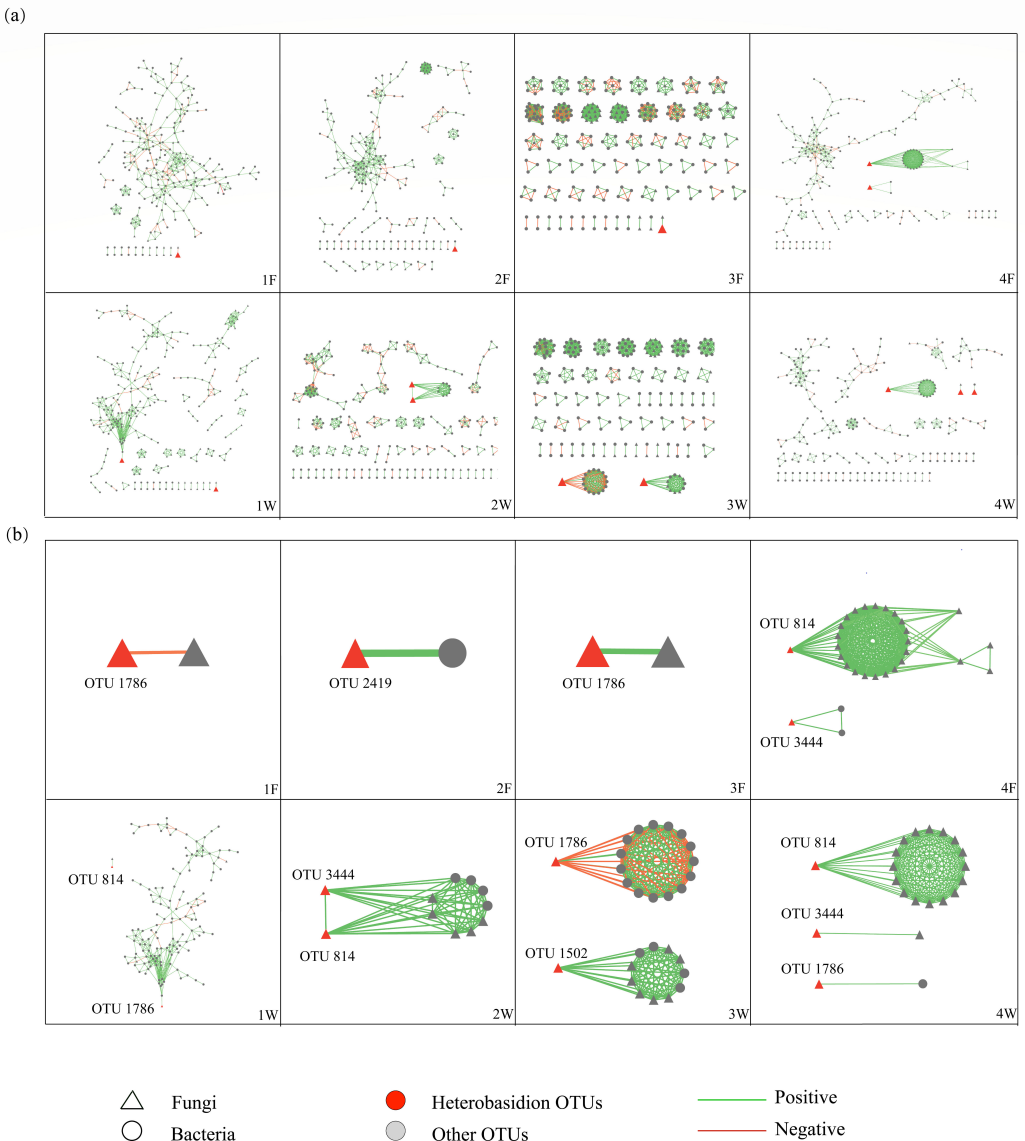
Hierarchy connection of myco- and microbiome networks in fruiting bodies and wood of decay classes. (**a**) Hierarchy network of the whole community. (**b**) Hierarchy network of *Heterobasidion*.

The network experienced a process of network fragmentation from the D1 class to the D3 class and reconstruction from D3 to D4 in both substrates (Fig. 3a). Abundant independent subnetworks were built in the D3 class of both materials. Interactions in the D3 class were conservative and occurred within the subnetwork community. Moreover, most subnetworks contained only intrakingdom interactions; 51 out of 58 were in the fruiting bodies, and 44 out of 55 subnetworks were only constructed by the same kingdom. The appearance of the most abundant subnetworks was accompanied by the highest community connectivity in the D3 class. A more complex network was reconstructed again in the D4 class of both substrates (Fig. 3a).

Intriguingly, *Heterobasidion* interactions were conserved throughout the decay process of both materials. It either held a binary connection with other OTUs or was mostly in an isolated subnetwork (Fig. 3a and b). This phenomenon was especially obvious in the fruiting bodies. *Heterobasidion* in D1W had the most complicated network and was the only *Heterobasidion* connection in more than three hierarchy levels. In addition, all the remaining *Heterobasidion* connections had only one hierarchy level except the one in D4F (Fig. 3b). Only a few negative connections were found between *Heterobasidion* and others, and they only occurred with *Heterobasidion* OTU01786. Two fungal OTUs were connected to OTU01786 in the fruiting bodies, and 17 bacterial OTUs were connected to OTU01786 in wood (Fig. 3b).

The other crucial difference between the fruiting bodies and wood was that wood had at least two active *Heterobasidion* OTUs in the community of each decay class, while in the fruiting body, only D4F had two *Heterobasidion* OTUs (Fig. 3b). OTU01786 was the only *Heterobasidion* OTU detected in D1F and D3F; moreover, OTU02419 was the only *Heterobasidion* OTU detected in D2F (Fig. 3b). Two OTUs had a high appearance in both materials: OTU01786 was found in D1F, D3F, D1W, D3W, and D4W; OTU00814 was found in D4F, D1W, D2W, and D4W; and OTU03444 and OTU814 were found in the last decay class of both materials. OTU2419 and OTU01502 only showed up in D2F and D3W separately (Table S4). *Heterobasidion* was only connected to one kingdom in the subnetwork of the fruiting bodies, while in that of wood, both bacteria and fungi were found in the *Heterobasidion* connecting subnetworks (Fig. 3b).

### Constant OTUs in the interkingdom network

OTUs that appeared in the D1 class and were constantly active until the D4 class were defined as constant OTUs. There were 112 constant OTUs found in the fruiting body, 85 of which were bacteria and 27 of which were fungi. In wood, 111 OTUs were found as constant OTUs; 96 were bacteria and 15 were fungi. The bacterial and fungal compositions of constant OTUs were similar in fruiting body and wood at the phylum level. Only seven and two identified phyla were recorded from bacteria and fungi, respectively, in the fruiting body and wood. One bacterial phylum was identified as WPS2, which was found only in the fruiting body, and one bacterial phylum, Dependentiae, was found only in wood (Table 1; Table S5).

**TABLE 1:** OTUs constantly and opportunistically appearing in the decay process

Constant (1-2-3-4)			Opportunistic							
Phylum	F.b.	Wood	Fruiting body decay class				Wood decay class			
	No.	No.	D1	D2	D3	D4	D1	D2	D3	D4
Bacteria										
Acidobacteria	9	6	5	4	11	6	3	6	4	3
Actinobacteria	18	24								
Bacteroidetes	7	5								
Dependentiae	0	1								
Firmicutes	2	4								
Proteobacteria	42	54								
Verrucomicrobia	6	2								
WPS2	1	0								
Total	85	96	26				16			
Fungi										
Ascomycota	20	11	1	2	3	2	2	4	1	4
Basidiomycota	7	3								
Undefined	0	1								
Total	27	15	8				11			
Grand total	112	111	34				27			

OTUs that appeared only once in a decay class were defined as opportunistic OTUs. There were 26 and 16 bacterial OTUs identified as opportunistic in the fruiting body and wood, respectively, and 8 and 11 fungal OTUs were found in the fruiting body and wood, respectively (Table 1; Table S6).

Based on the total OTUs, the fruiting bodies had 5,918 and 674 bacterial and fungal OTUs, and wood had 5,469 and 946 bacterial and fungal OTUs, respectively. The major proportion of OTUs were neither constant nor opportunistic (35; Ren et al., unpublished data).

### Environmental factors influencing community structure and function

Environmental factors influenced the interkingdom and intrakingdom interactions. As *Heterobasidion* contributed dominantly to the community structure and function, the five detected OTUs that actively participated in the interaction were also treated as five influencing factors, together with six other environmental factors, used in the Mantel test analysis. Alpha diversity, community structure, and functions that showed dominance or significant changes from both fungal and bacterial communities were analyzed based on their correlations with the influencing factors (Fig. S1).

**FIG 4:**
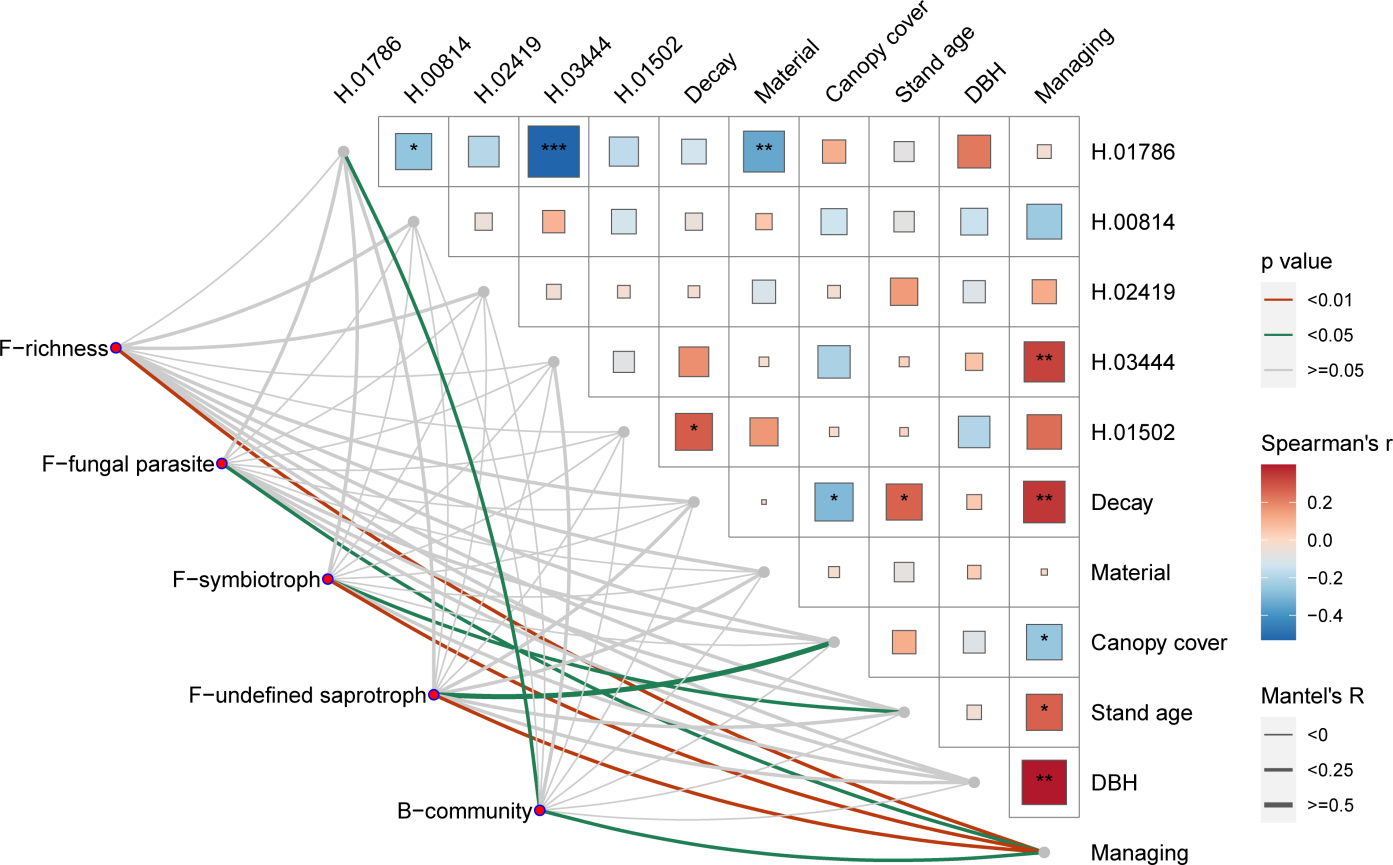
Drivers of micro- and mycobiome community compositions and functions. Pairwise comparisons of influencing factors (both from *Heterobasidion* OTUs and environmental factors) are shown, with a color gradient denoting Spearman’s correlation coefficient. The asterisk indicated the significance of Spearman’s correlation; “*” indicated *P* < 0.05, “**” indicated *P* < 0.01, and “***” indicated *P* < 0.001. *Heterobasidion* OTUs were represented by “H.” In the figure, for example, H.01786 represents *Heterobasidion* OTU01786. Taxonomic (including alpha diversity and community of fungi and bacteria) and function (based on the functions selected from previous studies) were related to each influencing factor by partial Mantel tests.

Surprisingly, forest management had the greatest impact on the macro- and micro-scales of the forest, and it was the most important driving factor for the microbial community and forest stand development. Significant correlations were shown between management and bacterial and fungal community or functional parameters (Fig. 4). Among the environmental parameters, management was negatively correlated with DBH, stand age, decay class, and abundance of OTU03444 but positively correlated with the canopy cover of the stand (Fig. 4).

*Heterobasidion* OTU01786 behaved as a second driving factor for community development. The bacterial community was positively correlated with both management intensity and OTU01786. OTU01786 was the only *Heterobasidion* OTU that correlated with the community. Moreover, OTU01786 was negatively correlated with OTU03444, OTU00814, and material. Study materials of fruiting bodies and wood were represented by values of “1” and “2,” so the negative correlation showed that a higher abundance of OTU01786 was present in the fruiting body.

The fungal community and its functions are more sensitive than those of bacteria. Fungi had more correlations with environmental factors than bacteria. Fungal richness and three fungal functions were correlated with some of the selected environmental factors. The identified microbial functions belonged to fungal parasites, fungal symbiotrophs, fungal saprotrophs, bacterial ureolysis, and plant pathogen-related bacteria (35; Ren et al., unpublished data). According to the ecological guild database (46), pathotrophs, symbiotrophs, and saprotrophs are the three trophic modes for classifying all fungi. Symbiotrophs represent the fungus group that “receives nutrients by exchanging resources with host cells,” and saprotrophs are fungi that “receive nutrients by breaking down dead cells.” Fungal parasites are classified as pathotrophs, which “receive nutrients by harming host cells.” The fungal function of symbiotrophs was positively correlated with both stand age and management. The fungal function of undefined saprotrophs was positively correlated with canopy cover and management. Moreover, fungal parasites and fungal richness were both positively correlated with management alone (Fig. 4). The bacterial community was the only bacterial parameter that correlated with environmental influencing factors (Fig. 4).

### Functions correlated with bacterial and fungal networks

**FIG 5:**
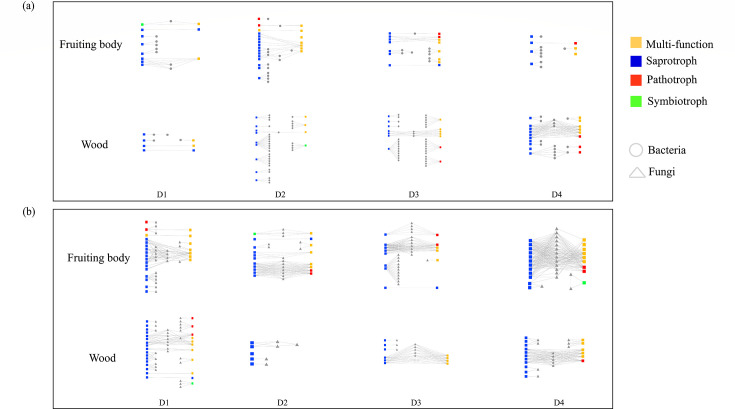
Correlation between functions and bacterial (**a**) and fungal communities (**b**).

The correlation between OTUs and functions indicated that the bacteria and fungi behaved differently in the decay process in the fruiting body and wood (Fig. 5). Clearly, the functional bacterial network had different complexities in the fruiting body and wood in the decay process (Fig. 5a). The function-bacteria correlation was simpler in the fruiting body but became more complex in wood with increasing levels of decay. On the other hand, function-fungi had a more complex network in the fruiting body, while in wood, the network was less complex (Fig. 5b).

The saprotroph function correlated with the most abundant bacteria and fungi in both the fruiting body and wood, with multiple functions that followed. Fewer bacteria correlated with saprotroph function in the decay process of the fruiting body, while the situation was the reverse in wood. More fungi are correlated with saprotrophs and other functions in the decay process of fruiting bodies and wood. Pathotroph-bacteria interactions became more abundant in wood with decay but less abundant in the fruiting body. Pathotroph-fungus interactions maintained the same abundance in the fruiting body. Rare fungi and bacteria interact with symbiotrophs. D2F and D2W were found in the symbiotroph-bacteria correlation; D2F, D4F, and D1W were found in the symbiotroph-fungi correlation.

## DISCUSSION

### The network and community

The combined analysis of interkingdom participants showed the overall picture, development, and contribution of the environment in changing the response and pattern of microbial communities in the *Heterobasidion* fruiting bodies and infected wood during the decay process. The results revealed that the interkingdom interaction was proportionally less than the intrakingdom. A previous study highlighted the importance of interkingdom interactions in revealing the resilience and resistance of microbial communities (20), during interactions with plants (22), and in ecosystem function prediction (21).

In the entire decay process, it was found that bacteria were more abundant and had more interactions than fungi, while the limited number of fungi may have a fundamental impact on the community as well as on *Heterobasidion* development. More fungal individuals showed higher connection degrees than bacteria. The microbial community in different materials developed into a fungi-dominated keystone species network. Additionally, the fungal community and functions seem to be more sensitive to environmental changes. This hypothesis was supported by previous studies, where fungi played an important role in shaping bacterial communities of decaying wood and influenced fungal physiology and behavior (49, 50). In this study, it is likely that bacteria behave as an adjustor in community development.

The microbial community network of wood and fruiting bodies experienced a process of fractionation and reconstruction (Fig. 1a and 3a). The networks in the D3 class were most fractionated, at the middle point of the change process, and were considered to have the least stable network communities. In this case, the stability of the community had a negative developing trend with bacterial activity. The decayed lignocellulose in this class may have contributed to the sudden change in nutritional environment that crushed down the community in the old nutritional mode (51). Correlating the abundance of active bacteria with the development of community stability, we also reasoned that the abundance of bacteria might be related to the instability of the community. The functional analysis of the bacterial community in the wood decay process also showed that nutrition and energy supply are essential functions (35). The result suggests that bacteria might influence fungal development by regulating community structure and nutrient supply.

In general, the stability of the community can be detected by combining positive and negative ratios (52) as well as the participating kingdoms of network interactions (53). Referencing the bacterial topology (35) and the fungal topology (Ren et al., unpublished data), the interkingdom topology has a similar indication of community activeness with bacteria, while the situation is quite different for fungi. Most of the community interactions were induced by bacteria, and the keystone OTUs in D3 of both materials also show the dominant participants of bacteria. Fewer fungal OTUs and fewer interkingdom interactions might be a possible reason for the lower activity. In nature, bacteria play the main role in microbial community structuring, while fungi mainly contribute to the function of the microbial community. This further proves that the sole intrakingdom analysis of the microbial community is incomplete and will not reveal the entire picture of the interacting microbiome. Interestingly, fungi have a more easily negative connection with bacteria than with fungi, and this might provide a clue for exploration for potential identification of *Heterobasidion* antagonistic and biocontrol agents.

### Core microbiome taxonomy

The results showed that a large amount of the microbiome participated in the decay process of wood tissue and fruiting bodies. However, only a small group plays a key role in the community and in the decay process.

Based on the analysis, a core microbiome defined by the overlap of three groups was identified to have a significant ecological role in this process. The first group is the keystone OTUs in the ecological interaction network; this particular group of microbiomes contains the most active individuals in the community. The high activeness suggests their dominating status in the decay process. The second group is the OTUs directly connected to *Heterobasidion*. Although *Heterobasidion* is quite conserved in the community, 68 OTUs from all samples were found to be directly or indirectly (at the third stage of the hierarchy level) connected to it. Two of the connections were negative. These connections indicate the relationship between *Heterobasidion* and the OTUs. A positive connection could indicate that the two OTUs share the same ecological niche or are mutualistic with each other; a negative connection shows that the two OTUs may have different niches or that they are competitors with each other. The third group is the OTUs that are constantly active in the decay process from the D1 class to the D4 class. This group of microbiomes can be constantly active regardless of environmental changes induced by decay, indicating its robustness in the community. Overall, 22 OTUs were found as core microbiomes in this study; 20 were from fungi, and 2 were from bacteria (Table S7).

Among the core microbiome, we referenced the published studies of OTUs identified at the species level. Some species have been recorded with clear potential as biocontrol agents. *Trichoderma polysporum* has been used as a biocontrol agent against weeds (54). It could be active as a mycoparasite and kill wood decay fungi in hyphal interactions. Some isolates from the species can produce active lytic enzymes that digest glucan and chitin and consume the hosts’ cytoplasmic content during parasitic activity. New peptide antibiotics, trichopolyn nitrate and trichopolyn hydrochloride (peptide), have been reported to be produced by *T. polysporum* (55). The antibiotic from the arctic isolate has also been reported to be inhibitory against the snow rot pathogen *Pythium iwayamai* (56). The species from *Trichoderma* have been shown to suppress infection by soil-borne root-infecting fungi (57), are able to suppress *Armillaria* development (58), and have been tested as a potential biocontrol agent for *Heterobasidion*. However, the suppression of *Heterobasidion* may not be as efficient as that of *Phlebiopsis gigantea*. The fast growth characteristic is believed to be the reason why the succession of slower-growing fungi is suppressed and reduced (59). The species *Chaetosphaeria vermicularioides* and *Botryobasidium obtusisporum* have been recorded as wood-inhabiting fungi or from decayed wood (60–65). Another record showed that *B. obtusisporum* is a lignin-degrading fungus (66). These two species show high tolerance under stress (67–69) and are common at higher decayed levels (70, 71).

The ecological functions of the remaining three species are not clear. *Lentinellus ursinus* has been recorded from both conifers and hardwoods (72). It was recorded as a white-rot fungus in one study (73). Several other studies record that it produces antibiotics against other microbiomes or predators (74–76). *Infundichalara microchona* is found in decayed and healthy pine wood (77–81). It was considered to be a saprotroph in two studies (30, 82). Kwaśna also reported it in the vascular wilt of poplar (70). However, it was recorded as an endophyte in another study (78). Various records were also found on *Sistotrema brinkmannii* as a wood decay fungus in several studies (83–86), and some isolates from the species produce high levels of lignocellulose (87) or cellulose (88) degrading enzymes. Moreover, it has been tested as a *Heterobasidion* biocontrol agent, although the antagonistic performance is lower than that of *P. gigantea*, and it is believed to be a successful colonizer after *P. gigantea* or *Heterobasidion* (86, 89).

The core microbiome identified in this study showed high overlap with the existing studies on tree pathogen and *Heterobasidion* antagonistic agent detection. The similarity among the core microbiome suggests that all the species have the potential to be pathogens or biocontrol agents. The diverse lifestyle allows for high adaptability and functional plasticity. By using the core microbiome selection method, the biocontrol or antagonistic agent can be selected from the group of OTUs that are directly connected, highly interacting with *Heterobasidion,* and constantly acting in the community. Different from previous studies, where biocontrol agents were selected based on the community differences between *Heterobasidion*-infected and noninfected trees, the current selection logic wasis based on the principle of microbiome interaction behaviour (15). In our approach, the core microbiomes may not be exclusively, antagonistic to *Heterobasidion*; they could also have other ecological functions, for example, stabilizing the *Heterobasidion*-created microbial community or supplying nutrition to the community. If the microbes are beneficial to *Heterobasidion*, suppressing this group of microbes would also suppress the development of *Heterobasidion* indirectly. The function and impact level of the microbial interaction would also be influenced by the environment (11), and adjusting the *Heterobasidion* infection microenvironment would definitely be able to regulate the infection result as well. Learning ofabout the microbes suppressing and supporting *Heterobasidion* in the community enlargesexpands the biocontrol agent selection range. Overall, the combination of both methods is believed to improve the biocontrol agent selection efficiency and accuracy.

### Impact of environmental factors

Based on the analysis, management background had the greatest impact on the fungal and bacterial communities. On a macro- scale, forest management regulates stand-level development for timber production and ecological sustainability (90). In our study, management had an impact on tree diameter, stand age, decay class, and canopy cover. Interestingly, management also showed significant impacts on the micro- scale in this study. Although the single -stand parameters had an impact on a few community functions, management had the most frequent impact on bacteria and fungi. Fungal symbiotrophs, fungal saprotrophs, and fungal parasites had positive correlations with management. Symbiotrophs, wood saprotrophs, and parasites were found to have positive correlations with management backgrounds. Forest management planning should take the microbial community into consideration for a healthy and sustainable forest stand. Old growth forest and retention trees were planned for higher biodiversity (91, 92), which could probably could be the reason for the positive correlation between stand age and symbiotroph abundance. The older stand with high biodiversity facilitated the survival of the symbiotrophic fungi. The existence of pathogens is more complicated, probably because of the high resilience of the old-growth tree and the high biodiversity competition. Canopy cover is the key parameter for stand structure, and weak trees lose competition in high -density stands (93). More deadwoods could be found in the high canopy cover stands; therefore, saprotrophs are positively connected to the canopy cover. However, management of the stand is able to increase fungal parasites as well as symbiotrophs and saprotrophs. The best management intensity level for a healthy microbial community might be predicted through a model based on metadata.

Forest management and *Heterobasidion* OTU01786 were also found to be positively correlated with the bacterial community. *Heterobasidion* OTU01786 is the most abundant *Heterobasidion* isolate, and it is possible that bacterial community development depends on the fungal community. OTU01786 is the most abundant *Heterobasidion* OTU and dominates the fruiting bodies and wood in all decay classes, according to a previous study (Ren et al., unpublished data). It is possible that this particular *Heterobasidion* OTU is the initial isolate in the infection. However, the decayed fruiting body and wood suggest that it could be the other *Heterobasidion* isolates that better fit the environment or provide better disease development. Energy and nutritional consumption do not seem to allow for simultaneous cohabitation of different isolates,; consequently, OTU01786, which fits the original condition, apparently lost its abundance. OTU01786 is negatively related to OTU03444 and OTU00814, and OTU03444 is negatively connected to management and was first found in the D2 class (Ren et al., unpublished data). Moreover, it was found to be the most abundant in D4F and D4W. It is possible that the divergent isolates divergent at the end of the decay process replaced OTU01786 with its better adaptation and dispersal function for seeking a suitable substrate to colonize.

According to Norway spruce decomposition research in the other environmental backgrounds of middle and southern Finland, the decomposition speed of Norway spruce has a slow start followed by accelerated growth in the middle, and a moderately slow ending in the range of 60–80 years of the whole decomposition process (36, 51). This trend corresponds to the network development trend of low activity and fewer modules in the D1 class, high activity and more modules in the D2 and D3 classes, and finally low activity and fewer modules again in the D4 class. This phenomenon indicates that the decay speed has a strong correlation with the network interaction performance in general. In this case, in addition to the environmental factors, which may vary greatly according to different geographical distributions, the changes in the substrate’s physico-chemical properties in the decay process might also have a strong correlation with the microbial community. From Hoppe’s study, total lignin, carbon concentration, C/N, and nitrogen concentrations are significantly different in diverse decay classes (94). Correlating these chemical parameters with the microbial community, we noticed that the fungal top degree distribution had the same increasing trend as the total lignin and a reverse trend to the carbon concentration in the wood decay process. Moreover, the bacterial keystone OTU numbers and bacterial modules had an opposite developing trend to the nitrogen concentration of the deadwood. This correlation further proves that the bacteria in the community are nutrition -related. In other studies, carbon percentage (C%), nitrogen percentage (N%), phosphorus (P), sulfur (S), and some metal concentrations (Mg, Mn, Ca), lignin concentration and C/N have significant differences among decay classes (95). Moreover, the top fungal degree had the same developing trend as the C%, Ca, and Mg concentrations. The P concentration had the same developing trend as the *Heterobasidion* relative abundance and edge number. It is reasonable to presume that P has significant nutritional value for *Heterobasidion* development.

Finally, the study revealed that bacteria had more participating individuals in the community, while fungi were most active, and had more connections in the community. Rare negative ecological correlations were found in fungi–fungi interactions but comparably more in fungi–bacteria interactions. The keystone OTUs were dominated by different kingdoms in the fruiting bodies and wood at the beginning of the decay process.

## Data Availability

The data analyzed in this study can be found in NCBI under Sequence Read Archive PRJNA992966 (Ren et al., unpublished data) and project number PRJNA800074, doi: 10.3389/fmicb.2022.864619 (36).

## SUPPLEMENTAL MATERIAL

Fig. S1 aem.01406-23-s0001.pdf Potential drivers of micro- and mycobiome community compositions and functions.

Table S1 aem.01406-23-s0002.xlsx Environmental factors considered in the Mantel test.

Table S2 aem.01406-23-s0003.xlsx Intra-kingdom and inter-kingdom interaction counts in each group.

Table S3 aem.01406-23-s0004.xlsx Keystone OTUs in each study group.

Table S4 aem.01406-23-s0005.xlsx OTUs in *Heterobasidion* hierarchy connections.

Table S5 aem.01406-23-s0006.xlsx OTUs are constantly active in fruiting bodies and wood.

Table S6 aem.01406-23-s0007.xlsx Opportunistic OTUs in each group.

Table S7 aem.01406-23-s0008.xlsx Core microbiome defined by the overlap of keystone OTUs, *Heterobasidion* directly connected OTUs, and constant active OTUs.

ASM does not own the copyrights to Supplemental Material that may be linked to, or accessed through, an article. The authors have granted ASM a non-exclusive, world-wide license to publish the Supplemental Material files. Please contact the corresponding author directly for reuse.
