# Supplementary material for: Interkingdom and intrakingdom interactions in the microbiome of Heterobasidion fruiting body and associated decayed woody tissues
Source: Appl Environ Microbiol. 2023 Nov 28;89(12):e01406-23. doi: 10.1128/aem.01406-23 (PMC10734517; doi:10.1128/aem.01406-23)
Supplement: Fig. S1 — Potential drivers of micro- and mycobiome community compositions and functions. [file aem.01406-23-s0001.pdf]

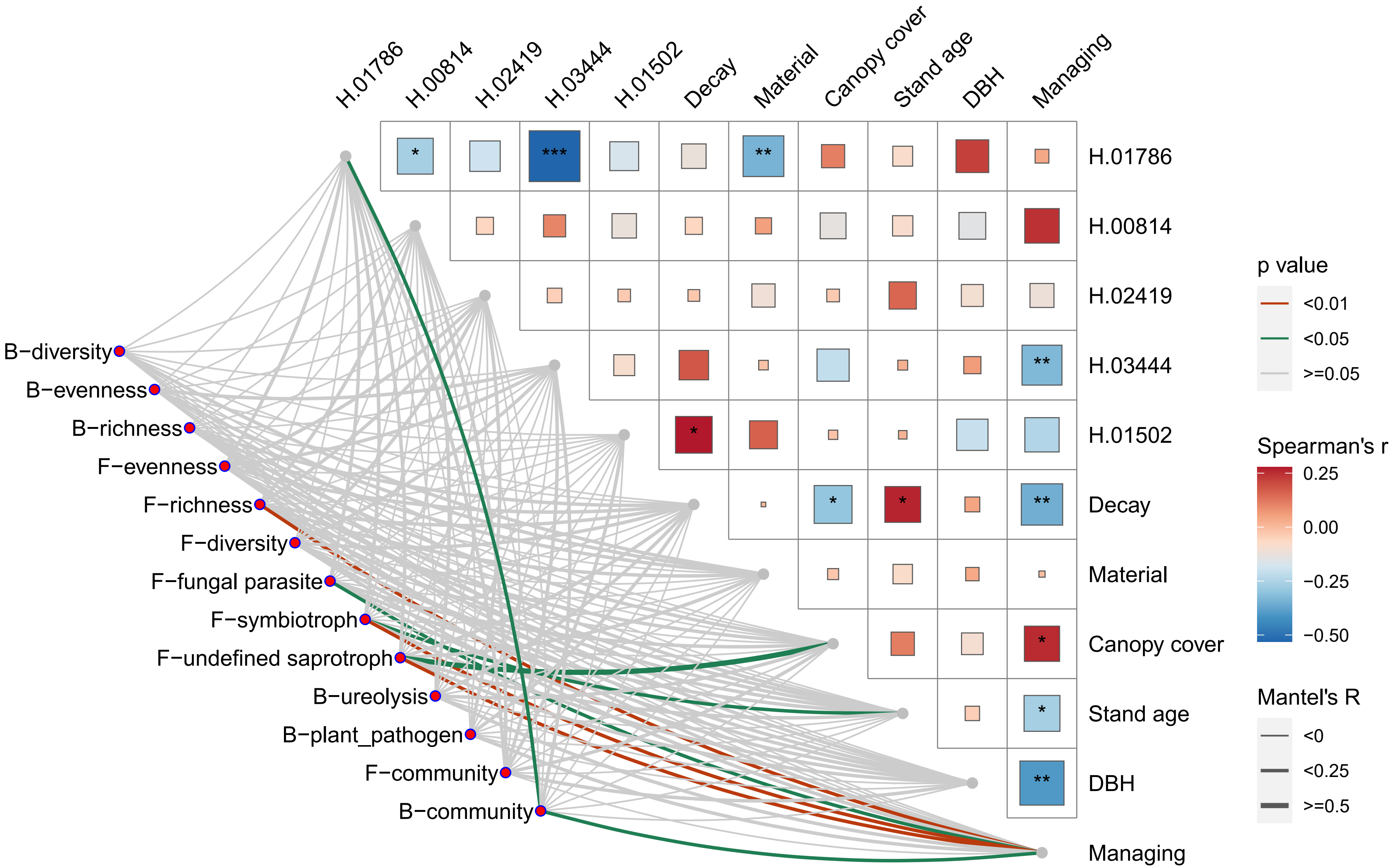


Supplementary Figure 1. Potential drivers of micro- and mycobiome community compositions and functions.
